# Do Attitudes towards Gender Equality Influence the Internalization of Ambivalent Sexism in Adolescence?

**DOI:** 10.3390/bs14090805

**Published:** 2024-09-11

**Authors:** Enrique Bonilla-Algovia, Concepción Carrasco Carpio, Rafael García-Pérez

**Affiliations:** 1 Department of Education, Distance University of Madrid (UDIMA), 28400 Madrid, Spain; enrique.bonilla@uah.es; 2 Department of Economics (Sociology), University of Alcalá (UAH), 28801 Madrid, Spain; 3 Department of Research and Diagnostic Methods in Education (MIDE), University of Seville, 41004 Sevilla, Spain; rafaelgarcia@us.es

**Keywords:** attitudes towards equality, gender inequality, hostile sexism, benevolent sexism, adolescence

## Abstract

Sexism during adolescence may hinder the attainment of gender equality and the eradication of violence against women. The aim of this research was to analyze the relationship between an individual’s view on gender equality and the internalization of ambivalent sexism, as well as to study the impact of different types of egalitarian attitudes (sociocultural, relational, and personal) on the levels of hostile and benevolent sexism among the adolescent population. A quantitative approach with a cross-sectional design was employed in this research. The sample group consisted of 1840 students (50.1% female and 49.9% male) in Compulsory Secondary Education in Castilla-La Mancha, Spain. The results show that adolescents who endorse pro-gender-inequality attitudes exhibit greater levels of hostile and benevolent sexism than their counterparts. Conversely, adolescents adopting pro-equality attitudes leads to lower levels of ambivalent sexism. In both male and female adolescents, egalitarian attitudes at the sociocultural, relational, and individual levels have a negative impact on the internalization of sexism. It is therefore crucial for both schools and families to prioritize the instruction of egalitarian values from early childhood, as this will aid in advancing a fairer and more equal society whilst eradicating sexist biases and violence against women.

## 1. Introduction

The World Health Organization [1] defines adolescence as a period of growth and development between childhood and adulthood, occurring from ages 10 to 19. However, Sawyer et al. [2] suggest that extending adolescence from 10 to 24 years of age would align better with current patterns and expectations of human development. This period of human life can be regarded as a crucial stage for psychosocial development, because in addition to the apparent physical changes, it is also characterized by numerous psychological and social changes [3,4,5]. Thus, adolescence is a critical developmental stage, although all stages of life are conducive to forming values that support gender equality and combat violence against women [6,7]. The support or opposition to gender equality among adolescents is a significant indicator of the future direction of society. As future adults, their views on gender equality and inequality can provide insights into the societal changes that may occur in the coming decades.

As stated by the United Nations Population Fund [8], gender equality is a fundamental human right that encompasses the balance between women and men in all spheres of social and personal life, including but not limited to economic independence and access to health and education systems. The Gender Equality Index assesses gender equality through a variety of factors, including work, money, knowledge, time, power, and health [9]. The Gender Inequality Index assesses gender-based disparities in the labor market, empowerment, and reproductive health [10]. The prevalence of violence against women serves as an additional indicator that societies remain far from achieving equality. This phenomenon represents one of the most extreme manifestations of gender inequality [9]. The concept of gender equality is founded upon the acknowledgment and elimination of material, cultural, and symbolic inequalities that impede progress across all domains of life. In alignment with the provisions set forth by the Convention on the Elimination of All Forms of Discrimination against Women (CEDAW), the attainment of gender equality requires not only the establishment of legal norms that guarantee equality before the law for women and men (formal equality), but also removing all barriers that prevent equality from becoming visible in fact (substantive equality) [11].

Gender equality, within the framework of the 2030 Agenda for Sustainable Development, must be included as one of the transversal goals to achieve human development worldwide [12]. In the context of the global partnership for sustainable development, the elimination of violence against women and girls has also become a global priority [13,14]. Consequently, various societies are implementing actions and policies that are aligned with the promotion of gender equality and the eradication of sexism and violence against women. Despite social support in recent decades, inequalities, gender roles, and sexist attitudes can be difficult to change. Heise et al. [15] illustrate the deep-rooted connection between gender norms and particular mind-sets that individuals learn in childhood, even though they are not necessarily permanent. This study aims to analyze whether attitudes towards equality can influence the internalization of sexism in adolescence.

Attitudes towards gender equality are related to people’s predisposition to equality. Consequently, they delineate a person’s position at the three levels of gender socialization [16]: sociocultural, relational, and personal. The first level refers to social gender roles and the distribution of domestic and family work; the second level refers to interpersonal relationships, including issues such as leadership, emotions, roles, and gender-based violence; and the third level refers to preferences, expectations, beliefs, and gender identity from a personal point of view. Thus, the positioning towards gender equality at the sociocultural, relational, and personal level allows for the differentiation of three attitudinal profiles [16]: *the egalitarian profile*, which implies recognition of the different forms of gender inequality and unequivocal support for egalitarian values; *the adaptive profile*, which implies a posture linked to political correctness, insofar as only the most explicit inequalities that are socially rejected are recognized; and *the unequal profile*, which implies non-recognition of gender inequality and the consequent reproduction of gender roles and patriarchal values, even in an unconscious manner.

Sexism, as defined by Glick and Fiske [17,18] using the ambivalence approach, involves two forms with opposing yet complementary sentiments (subjectively positive and negative) that strengthen each other instead of conflicting: hostile sexism and benevolent sexism. Hostile sexism involves overtly negative and coercive attitudes towards women, matching the forms of classical sexism, while benevolent sexism reflects subjectively positive attitudes—with a paternalistic and condescending tone—that stereotype and restrict the social role of women [17,18,19]. In this respect, despite the contrasting evaluations of women implied by the two forms of ambivalent sexism, research has consistently shown direct and positive correlations between them [20,21,22,23,24,25].

Ambivalent sexism is a multidimensional phenomenon, yet its two distinct forms share a common objective: the perpetuation and maintenance of an unequal gender structure. Consequently, hostile and benevolent sexism are associated, because they represent two sides of the same construct [17,18]. Sexist hostility is directed towards women who challenge traditional gender roles, whereas sexist benevolence involves a positive evaluation of women who accept these roles. Ambivalent sexism, which consolidates men’s power and limits women’s roles in the public and private fields [19], is an ideology of inequality. This is reflected in various studies conducted in recent decades. The concept of ambivalent sexism has been evaluated in a variety of cross-cultural contexts, with both forms of sexism being found to be related to gender inequality [20,21,22,23,24,26]. Therefore, empirical evidence shows that in countries with high levels of hostile and benevolent sexism, gender equality indexes tend to be lower [23,24].

Based on the results obtained from various national and international studies, it is emphasized that the adolescent population still shows gender biases, unequal beliefs, and sexist attitudes that reflect social inequalities between women and men [7,16,20,21,22,25,27,28,29,30,31,32,33,34]. In fact, research exploring cross-cultural differences in adolescents indicates that social changes are not uniformly affecting all contexts; furthermore, there appears to be a deceleration in gender equality [30]. In the specific case of ambivalent sexism, recent research findings suggest that sexist attitudes are deeply rooted in the adolescent worldview, with girls demonstrating a greater awareness of societal sexism than boys. Ayala et al. [27] discovered that, in a sample of adolescents from Spain, Portugal, Romania, Poland, Italy, and the United Kingdom, sexist attitudes persist and are more prevalent in boys, particularly with respect to hostile and benevolent sexism. Gil et al. [31] revealed that ambivalent sexism is prevalent among adolescents; furthermore, the study demonstrated that girls face social pressure to comply with behaviors conforming to socially accepted norms. In Castilla-La Mancha, Díaz and Sánchez [21] observed that boys exhibit significantly higher levels of hostile sexism than girls, whereas Carrasco et al. [29] found that boys score higher in all categories of sexism.

The academic literature suggests that adolescents, especially girls, living in more gender-equal environments generally show more egalitarian attitudes concerning gender roles [30]. This serves as a useful reference when designing educational programs that promote gender equality. According to previous studies [35,36], there is a negative correlation between gender awareness and neo-sexism in young people. This means that higher gender awareness and equality levels are linked to lower levels of neo-sexism and vice versa. Merma-Molina et al. [33], similarly, demonstrate that traditional gender roles heighten the likelihood of sexism during adolescence. Hence, they argue that promoting egalitarian roles and values can help prevent such attitudes. In samples of university students and the general population, Megías et al. [37] identified a positive correlation between myths about intimate partner violence against women and ambivalent sexism and a negative correlation between these variables and feminist ideology. Therefore, it seems that egalitarian or feminist education may be one of the most effective instruments for eradicating sexism and achieving a fully egalitarian society devoid of violence against women [6,26,38,39].

Achieving gender equality through education may be more difficult than expected, as social reality shows that, although it is necessary, it is not enough for educational institutions to be free from sexism [6]. Moreover, conscious efforts are required to counteract the influence of patriarchal society and establish principles that bring us closer to egalitarianism. In other words, educational institutions must collaborate with families to make equality a fundamental principle of socialization [38]. The educational system, based on coeducation and comprehensive sexuality education, must take action to prevent the perpetuation of gender stereotypes, sexist attitudes, and violence against women [38,39]. This transformation requires that educators receive training not only to identify gender inequalities but also to develop the essential tools to promote egalitarian attitudes among younger generations [26,40,41]. It is only when educators and families are aware of the persisting inequalities in multiple spheres of life that they will be able to promote equality and therefore take action to eliminate the barriers that hinder it.

Given the above, the objective of the present study is to examine the relationship between an individual’s view on gender equality and the internalization of ambivalent sexism, as well as to investigate the impact of different types of egalitarian attitudes (sociocultural, relational, and personal) on the levels of hostile and benevolent sexism among adolescents. The research hypotheses are as follows: firstly, that adolescents with egalitarian profiles will show lower levels of sexist attitudes than their peers (hypothesis 1); and secondly, that egalitarian attitudes at different levels will have a negative impact on the internalization of hostile and benevolent sexism in both boys and girls (hypothesis 2).

## 2. Method

### 2.1. Participants

This study included 1840 students (50.06% girls and 49.94% boys) in the third and fourth year of Compulsory Secondary Education in Castilla-La Mancha Autonomous Community (see Table 1). Age-wise, the study included students aged between 12 and 18 years, with an average age of 14.67 years (SD = 0.89). Out of all the participants, 52.77% were enrolled in the third year and 47.23% in the fourth year of Compulsory Secondary Education. The sample was representative of Castilla-La Mancha, with a confidence level of 95% and a margin of error of 2.2% for a population of around 40,000 students in the third and fourth grade. The students were selected using stratified random sampling, which included all five provinces (Albacete, Ciudad Real, Cuenca, Guadalajara, and Toledo) and districts of different sizes (rural, semi-urban, and urban) in the Autonomous Community.

### 2.2. Instruments

- *Adolescent Sexism Detection Scale* (ASD) [25]: Specifically designed to measure ambivalent sexism in adolescents, the ASD consists of twenty-six items divided into two dimensions. The first dimension evaluates attitudes related to hostile sexism using 16 items, while the second dimension evaluates attitudes towards benevolent sexism using 10 items. The response format is a Likert-type scale ranging from 1 (strongly disagree) to 6 (strongly agree), where higher scores indicate higher levels of ambivalent sexism. The reliability obtained in this study for the ASD was 0.93. The reliability for the two subscales was 0.91 for hostile sexism and 0.87 for benevolent sexism. The mean obtained in ASD was 1.91 (SD = 0.72)
- *School Doing Gender/students Scale* (SDG/s) [16]: The intention of this scale is to measure attitudes towards gender equality culture and coeducation among students. The scale has 30 items divided into three analytical levels: personal (10 items), sociocultural (10 items), and relational (10 items). The answer format comprises a Likert-type scale that offers five alternatives: 1 signifies total disagreement, while 5 indicates full agreement. Students can be categorized into three groups, based on their perspective on gender equality: those with an *unequal profile* (score ≤ 89); those with an *adaptive profile* (score between 90 and 119); and those with an *egalitarian profile* (score ≥ 120). The alpha coefficient obtained in this study indicated adequacy in the SDG/s (α = 0.92), and that was the case for its three levels: personal (α = 0.80), sociocultural (α = 0.81), and relational (α = 0.80). The average score obtained in the SDG/s was 133.72 (SD = 15.76).

### 2.3. Procedure

The sample of educational institutions was made accessible by the Women’s Institute and Regional Ministry of Education of Castilla-La Mancha. The randomly selected schools were informed of the study’s characteristics and contents through both telephone and email communication. The self-administered questionnaires were completed during the agreed-upon school hours with the management teams. The research team was present throughout the data collection process, clarifying the objectives and providing relevant instructions to the teachers and students. The tutor’s written informed consent formed the basis for the students’ participation. Respondents completed the questionnaire voluntarily and anonymously without any financial or other compensation. The University of Alcalá Research Ethics Committee approved the research project (CEI/HU/2019/39).

### 2.4. Analysis

The database was designed using SPSS statistical software (version 24.0). The levels of sexism were evaluated using the one-factor ANOVA model based on the students’ attitudes towards gender equality. Post hoc multiple comparisons were conducted to compare the differences between each pair of means among the various forms of sexism. Several multiple linear regression analyses were conducted to analyze the influence of attitudes towards equality on ambivalent sexism. The Introduction method was used. Finally, a classification model of the student body was created using the decision tree technique based on the level of sexism and the attitude towards gender equality. In the model, the missing values in both scales were replaced with the mean of the series. The Chi-square Automatic Interaction Detector (CHAID) was used as the growth method in the classification tree. For all the analyses, the results were interpreted as statistically significant according to values of *p* ≤ 0.05.

## 3. Results

Table 2 shows the average scores concerning hostile sexist attitudes based on students’ attitudes towards gender equality. On all the items that comprise the hostile sexism subscale, students with an unequal profile have considerably higher scores in contrast to the egalitarian and adaptive profiles (*p* < 0.001). The most significant differences between the three groups are found in item 23 (*Men should make the most important decisions in the couple’s life*; F = 394.36, *p* < 0.001) and item 9 (*Taking good care of the household is the responsibility of women*; F = 358.66, *p* < 0.001). On the other hand, the smallest differences are found in item 7 (*It is more natural for daughters and not sons to take care of their elderly parents*; F = 98.06, *p* < 0.001) and item 26 (*A man should bear affectionate, yet decisive, control over his wife*; F = 116.10, *p* < 0.001).

The mean differences in benevolent sexist attitudes are also significant based on the students’ attitudes towards gender equality (see Table 3). However, although students with an unequal profile score higher in all items, the ANOVA values are lower than those found for hostile sexism. The most significant mean differences are observed in item 21 (*Women are irreplaceable in the home*; F = 166.09, *p* < 0.001) and item 11 (*No one knows how to raise children better than women*; F = 103.43, *p* < 0.001). Regarding attitudes with smaller differences, item 1 (*Women are naturally more patient and tolerant than men*) is the only one where all profiles score higher than 3, thereby making the mean comparison statistic lower (F = 9.09, *p* < 0.001).

**Figure 1:**
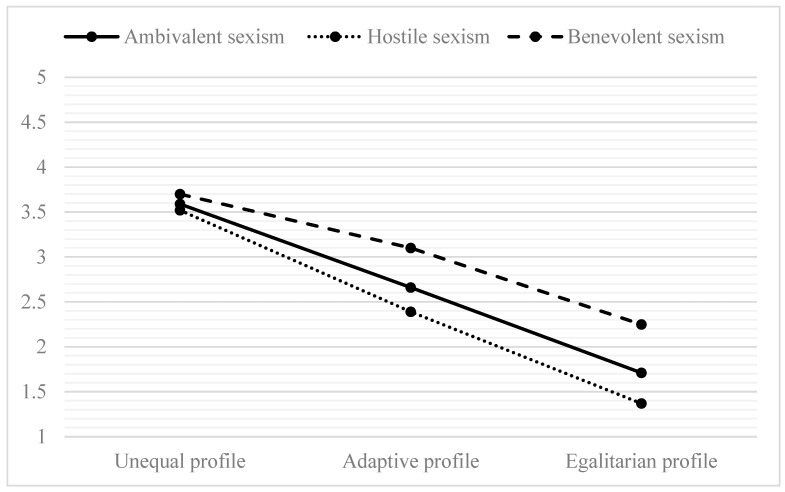
Levels of sexism according to students’ attitudes towards equality.

Figure 1 shows the distribution of students’ mean scores on the different forms of sexism according to their gender equality profile. On the one hand, students with an unequal profile (M = 3.59, SD = 0.95) show higher levels of ambivalent sexism compared to students with an adaptive profile (M = 2.66, SD = 0.62) and students with an egalitarian profile (M = 1.71, SD = 0.56) (F = 439.18, *p* < 0.001; Welch = 309.11, *p* < 0.001). On the other hand, for the two forms of ambivalent sexism, the results report that those with an egalitarian profile exhibit significantly less hostile sexism (M_egalitarian_ = 1.37, SD = 0.44; M_adaptive_ = 2.39, SD = 0.65; M_unequal_ = 3.52, SD = 0.97) (F = 738.83, *p* < 0.001; Welch = 353.31, *p* < 0.001) and less benevolent sexism (M_egalitarian_ = 2.25, SD = 0.91; M_adaptive_ = 3.10, SD = 0.81; M_unequal_ = 3.70, SD = 1.10) (F = 128.78, *p* < 0.001; Welch = 132.33, *p* < 0.001).

After confirming the presence of mean differences among the three profiles, post hoc multiple comparisons were conducted to compare the differences between each pair of means in the various types of sexism. The post hoc comparisons were performed using the Games–Howell test, since the assumption of homoscedasticity was not met in the homogeneity of variance test (*p* < 0.05). The results indicate that the differences between all pairs of means were statistically significant in the three types of sexism (see Table 4): Students with an unequal profile were significantly more sexist than those with egalitarian or adaptive profiles. Students with an adaptive profile were significantly more sexist than those with an egalitarian profile, and less sexist than those with an unequal profile. Finally, students with an egalitarian profile were significantly less sexist than their counterparts with either an egalitarian or adaptive profile.

**Figure 2:**
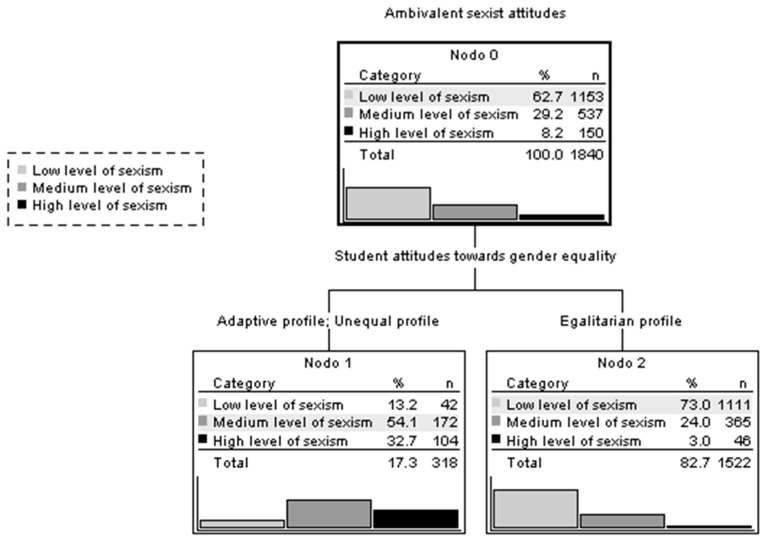
Tree diagram for the estimation of ambivalent sexism.

Additionally, a classification tree was created to further explore the influence of attitudes towards equality on ambivalent sexism (see Figure 2). For this purpose, scores on the ASD are grouped into three (dependent variable): a low level of sexism (score ≤ 2.00), medium level of sexism (score between 2.01 and 3.00), and high level of sexism (score ≥ 3.01). The independent variable is the attitudes of the students towards gender equality: egalitarian profile, adaptive profile, and unequal profile. The results suggest that attitudes supporting gender equality are associated with levels of ambivalent sexism: 73% of adolescents showing an egalitarian profile exhibit low levels of sexism, whilst 86.8% of adolescents with unequal or adaptive profiles display medium or high levels of sexism. It is noteworthy that only a small percentage of adolescents with an egalitarian profile (3%) demonstrate high levels of ambivalent sexism.

Finally, three linear regression analyses were conducted to investigate the impact of egalitarian attitudes on different forms of sexism—ambivalent, hostile, and benevolent. These three types of sexism serve as the dependent variables (see Table 5). The independent variables comprise attitudes towards equality at three levels—sociocultural, relational, and personal. All three statistical analyses show that egalitarian attitudes have an influential role in the regression models, with values ranging from 1.84 to 1.97 in the independence of errors test (Durbin–Watson) and between 2.50 and 3.02 in the collinearity test (IVF). Attitudes towards gender equality at the sociocultural, relational, and personal levels enable the prediction of the variance of ambivalent sexism (52.79% with negative regression coefficients; R = 0.727; ANOVA = 632.89, *p* < 0.001), hostile sexism (63.04% with negative regression coefficients; R = 0.794; ANOVA = 965.59, *p* < 0.001), and benevolent sexism (26.52% with negative regression coefficients; R = 0.515; ANOVA = 204.29, *p* < 0.001).

Linear regression analyses, disaggregating the sample by sex, indicate that girls’ ambivalent sexism is influenced by attitudes towards gender equality at the sociocultural level (β = −0.328, t = −9.86, *p* < 0.001), attitudes towards gender equality at the relational level (β = −0.340, t = −10.74, *p* < 0.001), and attitudes towards gender equality at the personal level (β = −0.164, t = −4.90, *p* < 0.001), explaining 52.06% of the variance (R = 0.722; ANOVA = 308.03, *p* < 0.001). For boys, ambivalent sexism is also influenced by attitudes towards gender equality at the sociocultural level (β = −0.273, t = −6.76, *p* < 0.001), attitudes towards gender equality at the relational level (β = −0.392, t = −10.61, *p* < 0.001), and attitudes towards gender equality at the personal level (β= −0.151, t = −3.68, *p* < 0.001), explaining 55.73% of the variance (R = 0.747; ANOVA = 335.32, *p* < 0.001). Along the same line, the regression analyses reflect that the levels of hostile and benevolent sexism are affected by all three types of attitudes towards gender equality (sociocultural, relational, and personal), with negative and significant coefficients in girls and boys (see Table 6).

## 4. Discussion

Adolescence, considered a crucial phase in the psychosocial growth of humans [3,4,5], is generally viewed as a significant time for internalizing or contending with sexist attitudes and unequal values that are present in the social context [6,7], as well as in the prevention of violence against women. However, although there have been advancements towards gender equality in recent decades, the academic literature indicates that sexism, in both its hostile and benevolent forms, remains prevalent across cultures [23,24,26] and among the adolescent population [20,21,22,25,27,29,31,34]. For these reasons, it is crucial to analyze the factors that are associated with the spread of sexism within the younger generations.

Previous national and international research has shown that sexism is linked to gender inequality [20,23,24,26] and to myths about intimate partner violence against women [37]. However, egalitarian attitudes should involve the recognition of inequality and the rejection of violence against women [16]. In this regard, as proposed in hypothesis 1, the findings of this research indicate that students with an egalitarian profile exhibit lower levels of ambivalent sexism, hostile sexism, and benevolent sexism compared to students with unequal or adaptive profiles. In contrast, adolescents with unequal profiles demonstrate the highest levels of all types of sexism. Consequently, empirical evidence indicates two directions: on the one hand, new forms of sexism can bolster inequality and impede gender awareness [26,35,36], and on the other hand, egalitarian gender attitudes can prevent the internalization of sexism during adolescence.

The cross-country studies conducted by Glick et al. [23,24] supported the theory that as national indicators of gender equality increase, the levels of ambivalent sexism decrease in both men and women. Recent studies indicate that adolescents in more egalitarian contexts tend to show more egalitarian gender attitudes [30] and that traditional adolescent gender roles are a significant predictor of sexist attitudes [33]. The regression analyses in the current study indicate that all three types of attitudes towards gender equality (sociocultural, relational, and personal) have an impact, with negative coefficients, on the internalization of hostile and benevolent sexist attitudes by adolescent boys and girls (hypothesis 2). Therefore, considering that boys tend to exhibit more sexist tendencies than girls [20,27,29,33], these results allow us to conclude that the assumption of egalitarian values at the sociocultural, relational, and personal levels is related to a reduced acceptance of sexism in adolescence, especially in relation to a lower internalization of the most hostile and coercive sexist attitudes, not only in girls, but also in boys.

One function of sexism is to legitimize gender inequalities in society [26]. However, egalitarian attitudes may hinder the reproduction of ambivalent sexism, and they are, consequently, an impediment to the perpetuation of gender inequality among new generations, according to this research. Carrascosa et al. [42] successfully reduced the levels of ambivalent sexism in teenagers through an intervention program in educational centers. Mobile-based psychoeducational applications have also been effective in reducing sexism, according to Navarro-Pérez et al. [43]. Since the sociocultural context can impact gender attitudes in adolescence [30], and gender norms can be blocked and transformed [15], efforts to promote equality in places where socialization takes place—specifically families and educational institutions [6,38,39]—should not be overlooked.

This research has limitations that must be acknowledged. Firstly, the cross-sectional design and correlational analyses used in this study allowed us to examine the different relations between attitudes towards equality and ambivalent sexism at a specific stage of adolescence. Nevertheless, this methodological paradigm limited our ability to determine whether these relations persist or vary over time. Secondly, although egalitarian attitudes seem to hinder the internalization of sexism during adolescence, the regression analyses do not permit the establishment of causal relationships between the study variables. Third and finally, the findings cannot be generalized to other cultures, as conceptions of gender equality and inequality may vary across different geographical contexts.

With regard to the prospective implications, in the future, it would be advisable to replicate this research with longitudinal samples to analyze if the impact of egalitarian attitudes on sexism persists beyond adolescence. Likewise, it would be beneficial to implement intervention programs that specifically promote gender equality and feminism during adolescence, analyzing the impact of these programs through a pre–post evaluation. It would be advantageous for future research to integrate cross-cultural approaches to determine whether the impact of egalitarian attitudes is consistent or variable depending on the cultural context. Additionally, it is strongly recommended that successful educational initiatives that reduce ambivalent sexism be widely shared among teachers, so that they may implement them in their respective schools.

In conclusion, one of the fundamental principles of the educational system is that it should prioritize the advancement of gender equality and the prevention of violence against women [6,38,39,40,41], particularly in contemporary times, as it is integral to achieving the Sustainable Development Goals [12,13,14]. Incorporating these objectives into educational contexts requires adopting a perspective on societal issues and addressing them through schools [40], although the responsibility should be shared with families [6,38]. Thus, it is crucial that initiatives and efforts to promote gender equality education remain consistent, as they can play an essential role in preventing the perpetuation of sexism and bringing society closer to real and substantive equality.

## Figures and Tables

**Table 1:** Sample characteristics.

	*n*	%	Mean (SD)
**Age range**			
12–14 years	848	46.19	
15–18 years	988	53.81	
**Mean age**			14.67 (0.89)
**Sex**			
Boys	893	49.94	
Girls	895	50.06	
**Educational level**			
Third grade	970	52.77	
Fourth grade	868	47.23	
**Nationality**			
Spanish	1675	91.03	
Foreign	165	8.97	
**Province**			
Albacete	177	9.62	
Ciudad Real	516	28.04	
Cuenca	145	7.88	
Guadalajara	316	17.17	
Toledo	686	37.28	
**Size of municipality area**			
Rural	100	5.43	
Semi-urban	740	40.22	
Urban	1000	54.35	

**Table 2:** Levels of hostile sexism according to students’ attitudes towards equality.

Items	Unequal Profile M (SD)	Adaptive Profile M (SD)	Egalitarian Profile M (SD)	F
2. The most suitable place for the woman is her home with her family.	3.41 (1.56)	2.65 (1.42)	1.56 (1.00)	145.14 ***
4. Women are weaker than men in every respect.	3.68 (1.49)	2.58 (1.36)	1.52 (0.98)	162.42 ***
5. Women staying at home would be a good way to combat unemployment.	2.91 (1.58)	1.75 (1.06)	1.10 (0.43)	254.68 ***
7. It is more natural for daughters and not sons to take care of elderly parents.	3.06 (1.61)	2.46 (1.25)	1.55 (1.05)	98.06 ***
9. Taking good care of the household is the responsibility of women.	3.42 (1.75)	2.00 (1.09)	1.14 (0.49)	358.66 ***
10. Women should be put in their place so that they do not dominate the man.	3.71 (1.84)	2.18 (1.21)	1.27 (0.74)	242.68 ***
12. Women are manipulative by nature.	3.85 (1.69)	2.57 (1.41)	1.39 (0.82)	255.48 ***
14. A man should be the main source of income for his family.	3.89 (1.61)	2.72 (1.34)	1.38 (0.80)	326.80 ***
16. The husband is the head of the family, and the wife should respect his authority.	3.51 (1.65)	2.21 (1.12)	1.19 (0.60)	351.47 ***
18. It is not proper for men to be in charge of household chores.	4.00 (1.59)	2.59 (1.33)	1.43 (0.95)	221.53 ***
19. Women reason worse than men.	2.86 (1.68)	2.37 (1.16)	1.33 (0.74)	202.28 ***
20. Men are more qualified than women for public affairs (e.g., politics, business, etc.).	4.15 (1.67)	2.59 (1.35)	1.38 (0.87)	276.87 ***
22. Women who work outside the home neglect their families.	3.24 (1.65)	1.96 (1.01)	1.19 (0.57)	261.14 ***
23. Men should make the most important decisions in the couple’s life.	3.69 (1.68)	2.20 (1.13)	1.19 (0.57)	394.36 ***
25. A woman should be willing to sacrifice herself for her husband’s professional success.	2.91 (1.84)	2.28 (1.20)	1.34 (0.74)	171.49 ***
26. A man should bear affectionate, yet decisive, control over his wife	4.00 (1.72)	3.18 (1.48)	1.94 (1.34)	116.10 ***

Note: *** *p* ≤ 0.001.

**Table 3:** Levels of benevolent sexism according to students’ attitudes towards equality.

Items	Unequal Profile M (SD)	Adaptive Profile M (SD)	Egalitarian Profile M (SD)	F
1. Women are naturally more patient and tolerant than men.	3.37 (1.70)	3.64 (1.33)	3.20 (1.46)	9.09 ***
3. Affection is more important to women than to men.	3.49 (1.63)	3.17 (1.38)	2.30 (1.41)	48.72 ***
6. Women are better gifted than men at pleasing others (being attentive to what they want and need).	3.63 (1.56)	3.21 (1.36)	2.09 (1.30)	93.53 ***
8. Because of their greater sensitivity, women are more compassionate than men towards their partner.	3.77 (1.68)	3.14 (1.30)	2.55 (1.39)	30.51 ***
11. No one knows how to raise their children better than women.	4.37 (1.70)	3.28 (1.58)	2.12 (1.40)	103.43 ***
13. Women have a greater capacity to forgive their partner’s faults than men.	3.86 (1.80)	2.98 (1.36)	2.37 (1.37)	37.58 ***
15. For a man, a fragile woman has a special charm.	3.45 (1.68)	2.97 (1.44)	1.98 (1.23)	78.64 ***
17. Women naturally possess a greater sensitivity than men.	4.03 (1.64)	3.37 (1.38)	2.46 (1.44)	58.68 ***
21. Women are irreplaceable in the home.	3.97 (1.71)	2.68 (1.43)	1.56 (1.06)	166.09 ***
24. By nature, women are better endowed than men to bear suffering.	3.06 (1.80)	2.52 (1.41)	1.88 (1.29)	36.13 ***

Note: *** *p* ≤ 0.001.

**Table 4:** Post hoc analysis of sexism according to students’ attitude towards equality.

	Students’ Profile (I)	Students’ Profile (J_1_)	Difference between Means (I-J_1_)	Students’ Profile (J_2_)	Difference between Means (I-J_2_)
Ambivalent sexism	Unequal profile	Adaptive profile	0.92 ***	Egalitarian profile	1.88 ***
	Adaptive profile	Unequal profile	–0.92 ***	Egalitarian profile	0.96 ***
	Egalitarian profile	Unequal profile	−1.88 ***	Adaptive profile	–0.96 ***
Hostile sexism	Unequal profile	Adaptive profile	1.12 ***	Egalitarian profile	2.15 ***
	Adaptive profile	Unequal profile	−1.12 ***	Egalitarian profile	1.02 ***
	Egalitarian profile	Unequal profile	−2.15 ***	Adaptive profile	−1.02 ***
Benevolent sexism	Unequal profile	Adaptive profile	0.60 **	Egalitarian profile	1.45 ***
	Adaptive profile	Unequal profile	−0.60 **	Egalitarian profile	0.85 ***
	Egalitarian profile	Unequal profile	−1.45 ***	Adaptive profile	–0.85 ***

Note: ** *p* ≤ 0.01; *** *p* ≤ 0.001.

**Table 5:** Linear regression for prediction of sexism.

	B	SE	Standardized B	t	*p*	CI 95%
**Ambivalent sexism:**						
Constant	6.175	0.102		60.40	0.000	5.975/6.376
Attitudes towards equality at the sociocultural level	−0.039	0.003	−0.318	−11.37	0.000	−0.046/−0.032
Attitudes towards equality at the relational level	−0.041	0.003	−0.349	−13.21	0.000	−0.047/−0.035
Attitudes towards equality at the personal level	−0.017	0.004	−0.128	−4.43	0.000	−0.024/−0.009
**Hostile sexism:**						
Constant	6.028	0.085		70.82	0.000	5.861/6.195
Attitudes towards equality at the sociocultural level	−0.039	0.003	−0.341	−13.77	0.000	−0.045/−0.034
Attitudes towards equality at the relational level	−0.036	0.003	−0.322	−13.81	0.000	−0.041/−0.031
Attitudes towards equality at the personal level	−0.025	0.003	−0.208	−8.12	0.000	−0.031/−0.019
**Benevolent sexism:**						
Constant	6.410	0.173		37.03	0.000	6.071/6.750
Attitudes towards equality at the sociocultural level	−0.039	0.006	−0.231	−6.62	0.000	–0.050/−0.027
Attitudes towards equality at the relational level	−0.050	0.005	−0.311	−9.45	0.000	−0.060/−0.039
Attitudes towards equality at the personal level	−0.003	0.006	−0.015	−0.40	0.688	−0.015/0.010

Note: B = unstandardized coefficient; SE = standard error; standardized B = standardized coefficient; t = Student’s T; *p* = significance; CI 95% = confidence interval for B at 95%.

**Table 6:** Linear regression by sex.

	R	F	B	SE	Standardized B	t
**Hostile sexism (girls):**						
Constant			6.834	0.170		40.13 ***
Attitudes towards equality at the sociocultural level	0.751	367.14 ***	−0.049	0.004	−0.380	−12.00 ***
Attitudes towards equality at the relational level			−0.038	0.004	−0.287	−9.50 ***
Attitudes towards equality at the personal level			−0.030	0.005	−0.199	−6.23 ***
**Hostile sexism (boys):**						
Constant			5.971	0.113		52.76 ***
Attitudes towards equality at the sociocultural level	0.805	489.99 ***	−0.033	0.004	−0.289	−8.04 ***
Attitudes towards equality at the relational level			−0.040	0.004	−0.362	−11.00 ***
Attitudes towards equality at the personal level			−0.027	0.004	−0.231	−6.31 ***
**Benevolent sexism (girls):**						
Constant			9.429	0.364		25.92 ***
Attitudes towards equality at the sociocultural level			−0.050	0.009	−0.223	−5.69 ***
Attitudes towards equality at the relational level	0.577	141.58 ***	−0.076	0.009	−0.334	−8.94 ***
Attitudes towards equality at the personal level			−0.027	0.010	−0.104	−2.63 ***
**Benevolent sexism (boys):**						
Constant			6.032	0.212		28.46 ***
Attitudes towards equality at the sociocultural level	0.534	106.38 ***	−0.030	0.008	−0.199	−3.89 ***
Attitudes towards equality at the relational level			−0.051	0.007	−0.352	−7.49 ***
Attitudes towards equality at the personal level			−0.004	0.008	−0.025	−0.47

Note: R = coefficient of determination; F = ANOVA; B = unstandardized coefficient; SE = standard error; Standardized B = standardized coefficient; t = Student’s T; *** *p* ≤ 0.001.

## Data Availability

Data are unavailable, because they belong to Institute for Women of Castilla-La Mancha.
